# Synthesis of Mg–Al Alkoxide/Ionic Liquid Particles as Heterogeneous Catalysts for Ring-Opening Polymerization of ε‑Caprolactone

**DOI:** 10.1021/acsomega.6c02540

**Published:** 2026-07-03

**Authors:** Michal Navrátil, Marwa Rebei, Darina Smržová, Anna Vykydalová, Petr Bezdička, Martin Kormunda, Natalija Murafa, Zuzana Walterová, Lívia Kanizsová, Hynek Beneš, Petra Ecorchard

**Affiliations:** † 112895 Institute of Inorganic Chemistry of the Czech Academy of Sciences, Husinec-Řež č. P. 1001, Řež 250 68, Czech Republic; ‡ 86879 Institute of Macromolecular Chemistry of the Czech Academy of Sciences, Heyrovského nám. 2, Prague 6 162 00, Czech Republic; § Polymer Institute, Slovak Academy of Sciences, Dúbravská Cesta 9, Bratislava 845 41, Slovakia; ∥ Faculty of Science, Jan Evangelista Purkyně University in Ústí Nad Labem, Pasteurova 3632/15, Ústí Nad Labem 400 96, Czech Republic

## Abstract

The widespread use of biodegradable plastics, especially polyesters, in biomedicine, packaging, and agriculture requires the investigation of efficient heterogeneous catalysts that are nontoxic and easily recyclable. Herein, we report solvolytic and solvothermal methods to synthesize bimetallic Mg–Al alkoxides (BMA) and their subsequent modifications using the phosphonium-based ionic liquid trihexyltetradecylphosphonium bis­(2,4,4-trimethylpentyl)­phosphinate (IL-PO_2_). The successfully modified Mg–Al alkoxide/ionic liquid particles were explored as a heterogeneous initiating/catalyzing system for the ring-opening polymerization (ROP) of ε-caprolactone. Morphological and structural studies were conducted in order to explore the properties in detail and to better understand the differences from layered double hydroxides modified with IL-PO_2_, which had already been applied to ROP. The thermal stability was confirmed up to 170 °C, i.e., sufficient for ROP. The water content was also determined by thermogravimetric analysis, and it was found that water is present as surface water and is incorporated into the structure. The explanation for the very good catalytic activity was possible after a model reaction with a higher concentration of catalysts, 10% for BMA-2-IL-PO_2_, which confirmed that IL-PO_2_ also actively participates in ROP.

## Graphical abstract

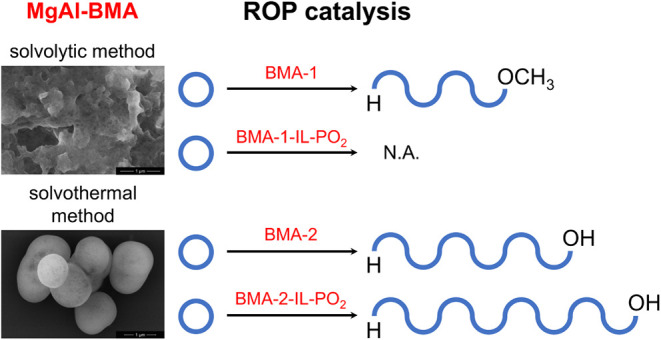

## Introduction

Layered double hydroxides (LDHs) enable the immobilization of catalytically active species and are therefore widely used in heterogeneous catalysis due to their enhanced activity, selectivity, stability, and recyclability compared to homogeneous catalysts.^1^ LDHs modified with ionic liquids (ILs) have also been investigated as heterogeneous initiators/catalysts for ring-opening polymerization (ROP) of cyclic lactones to produce biodegradable polyesters.^2–4^ These polymers are used in medical, food-packaging, or agricultural applications, where organometallic catalysts should be avoided. ILs immobilized on LDH surfaces facilitate polymerization and tune surface properties, such as hydrophilicity and molecular weight.^2–4^

The catalytically active LDH particles can be easily separated from the polymer and reused, because they are not covalently bonded to the polyester chain. The high hydrophilicity and the presence of intercalated water molecules are the main drawbacks of LDH particles, which lead to the formation of low-molar mass polyesters exhibiting insufficient end-use properties. Therefore, either their intensive/vacuum drying^2^ or their high-temperature treatment (e.g., calcination)^3,4^ prior to use is usually required. However, the first is an energy-demanding step, while the second leads to the destruction of the LDH layered structure. It is highly desirable to find novel water-free analogues of traditional LDH particles.

Such a water-free material with desired properties could be bimetallic alkoxides (BMA), a group of materials on the border between inorganic and organic chemistry. The inorganic part of BMA consists of two different metal cations, and the organic part of BMA is made up of alkoxide anions. Additionally, the structure can contain solvated or coordinated molecules of alcohols that saturate the metal cations’ coordination environment and stabilize the BMA structure.^5^

While the brucite-like structure of LDH is well known, the universal structure of BMA has not been determined. Two types of BMA are reported—discrete molecules and materials. The compositions of BMA are mostly transition-metal cations, and the structure can be confirmed by single-crystal X-ray diffraction, e.g., the structures of Mg_1.2_Ti_2.8_(OEt)_13.6_·2.4EtOH and Mg_2_Zr_2_(OPr)_12_(PrOH)_4_.^5,6^ Additionally, the structures can contain solvent molecules (tetrahydrofuran (THF) in Li_2_Zr_2_(OPr)_10_(THF)_2_) or auxiliary anions (acetylacetonate (acac) in Co_2_Zr_2_(OEt)_10_(acac)_2_).^6^ The X-ray structures corroborate two bonding modes of the alkoxide (terminal and bridging).

On the other hand, the structure of material BMA has not been well discovered. These materials were prepared as analogues of LDHs and are believed to exhibit a similar structure. In particular, there are positively charged layers containing metal cations, and the interlayer spaces are filled with compensating anions and solvent molecules.^7^

BMAs can be synthesized from LDHs. Siri-Nguan reported hydroxide/alkoxide-containing materials referred to as alkoxide-intercalated LDH derivatives. They are typically obtained by equilibrium-driven anion exchange. For example, nitrate-intercalated MgAl-LDH suspended in THF reacted with alkali metal alkoxides to exchange nitrate for alkoxide. X-ray powder diffraction (XRPD) confirmed changes in the hydrotalcite structure and the formation of alkali metal nitrates as byproducts.^8^ In ethylene glycol, alkoxide deprotonation led to intercalated ethylene glycolate species. Full conversion of nitrate to ethylene glycolate was achieved after 12 h at 120 °C using potassium *tert*-butoxide as the base.^9^

BMAs can also be prepared via coprecipitation. Similar to LDH synthesis, nitrate, chloride, sulfate, or acetate salts are reacted with alcoholic solutions of metal hydroxides. However, the products contain hydroxide anions and solvated water.^10–12^ Varga et al. modified the coprecipitation approach to a solvolytic method, where only alkoxide species react in alcoholic media, minimizing the water content. The resulting BMAs were stable in air for up to 9 months.^7^

In contrast, the solvothermal method uses a different synthetic approach. The reaction proceeds at 180 °C under pressure in an autoclave containing a glycerol/isopropyl alcohol mixture, without the addition of a base. Nickel­(II)–iron­(III) glycerolate, prepared from nitrate hydrates, formed spherical particles with a rough surface.^13^ The main drawback is the water introduced by hydrated precursors, although the high-temperature synthesis yields thermally stable products.

Water strongly influences the structural stability. Water forms stronger hydrogen bonds than alcohols, which promotes the formation of crystalline materials, similar to LDHs. However, alkoxides are susceptible to hydrolysis, and water induces BMA degradation. Both effects were utilized in the preparation of continuous oriented films, where a smooth BMA-based film was hydrolyzed into an LDH-based film.^12,14^

The aim of this study was to prepare pristine and IL-modified MgAl-BMAs using different synthetic routes and evaluate their catalytic performance in the ring-opening polymerization (ROP) of cyclic esters, especially ε-caprolactone (εCL). BMA was synthesized either by a solvolytic method using volatile alcohols (ethanol or methanol) or by a solvothermal method using glycerol. Subsequently, both procedures were also carried out in the presence of trihexyltetradecylphosphonium bis­(2,4,4-trimethylpentyl)­phosphinate (IL-PO_2_). The solvolytic method generally forms layered BMA structures, often modified with IL through a one-pot approach. The prepared materials were characterized to investigate their structure, residual water content, and thermal stability. Finally, the IL-PO_2_-modified BMA was used as a heterogeneous initiator/catalyst for microwave-assisted ROP of εCL. The resulting polycaprolactone (PCL) was analyzed by size exclusion chromatography (SEC) and MALDI-TOF mass spectrometry to determine its structure and polymerization initiation mechanism.

## Experimental Part

All syntheses applying the solvolytic method were performed under argon using standard Schlenk techniques. Solvothermal synthesis was performed in autoclaves (Col-Int Tech) with a Teflon liner. Methanol, ethanol, isopropyl alcohol, and glycerol (all A.G.) were purchased from Lach-ner (Czech Republic). Additionally, methanol and ethanol were dried with molecular sieves and stored under argon. Magnesium ethoxide and aluminum isopropoxide were manufactured by Merck, stored in a desiccator with SiO_2_, and ground in an agate mortar before use. Sodium metal (pure) was obtained from Sigma-Aldrich; its surface was cut off before use. The freshly prepared sodium ethoxide was synthesized from sodium metal and ethanol. Magnesium nitrate hexahydrate (A.G.) and aluminum nitrate nonahydrate (A.G.) were obtained from Roth. Trihexyltetradecylphosphonium bis­(2,4,4-trimethylpentyl)­phosphinate (IL-PO_2_, >95%) was purchased from IoLiTec (Germany) and used as received. Commercially available ε-caprolactone (εCL, Merck, 97%) was purified by vacuum distillation and stored under argon prior to use.

### Synthesis of BMA-1 by the Solvolytic Method

The synthesis was based on a method published by Varga et al.^7^ A 250 mL Schlenk flask was oven-dried (220 °C, 1 h) and afterward equipped with a magnetic stirrer, secured, and flushed with argon. Magnesium ethoxide (2.865 g; 25.0 mmol; 2.0 equiv) and aluminum isopropoxide (2.301 g; 11.3 mmol; 0.9 equiv) were mixed in the flask and suspended in 100 mL of dry methanol (see Scheme 1). In a 50 mL flask (under an argon atmosphere), 30 mL of ethanol (see Scheme 1) was introduced and cooled in a water bath, and sodium metal (1.150 g; 50.0 mmol; 4.0 equiv) was added in several portions. A sodium ethoxide solution was transferred to the suspension of Mg and Al alkoxides. The resulting mixture was stirred overnight at RT upon the formation of a fine suspension. After sedimentation, the excess liquid was sucked with a cannula and 50 mL of fresh solvent was added. The mixture was stirred for 1 h, and then the suspension sedimented. This washing process was repeated 3 times. The final mixture was stored as a suspension at 4 °C.

**Scheme 1:**
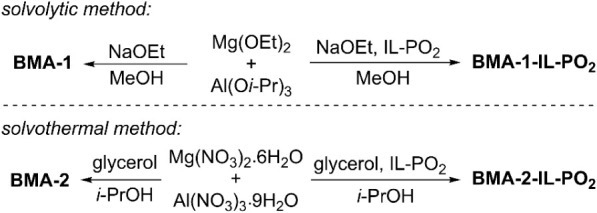
Preparation of BMAs via Solvolytic and Solvothermal Methods, Including Their Modification with IL-PO_2_ (Me = methyl, Et = ethyl, *i*-Pr = isopropyl)

### Modification of Solvolytically Prepared BMA-1 by IL-PO_2_

In an oven-dried (220 °C, 2 h) 250 mL Schlenk flask, an IL-PO_2_ was dried (5.00 g; 1 g of IL-P/5 mmol of Mg^2+^) at elevated temperature (150 °C) and under reduced pressure (200 Pa). Then, magnesium ethoxide (2.865 g; 25.0 mmol; 2.0 equiv) and aluminum isopropoxide (2.301 g; 11.3 mmol; 0.9 equiv) were added, and the flask’s contents were mixed with 100 mL of dry methanol (see Scheme 1). After the Mg- and Al-alkoxides became suspended and the IL-PO_2_ dissolved, a fresh solution of sodium ethoxide was added to the mixture. The sodium solution was prepared by dissolving sodium (1.150 g; 50 mmol; 4 equiv) in 30 mL of dry ethanol, followed by cooling in a water bath. The resulting reaction mixture was stirred overnight (∼16 h) at RT. The final solid was once washed with 50 mL of dry methanol. Similarly to the parental BMA-1, the modified materials were stored as suspensions at 4 °C.

### Synthesis of BMA-2 by the Solvothermal Method

A modified one-step synthetic procedure published by Wang was employed.^13^ Magnesium nitrate hexahydrate (0.8228 g; 3.2 mmol; 2 equiv) and aluminum nitrate nonahydrate (0.5998 g; 1.6 mmol; 1 equiv) were placed into a Teflon liner and dissolved in 126 mL of isopropyl alcohol. After stirring for 30 min, 25 mL of glycerol was added, and the mixture was stirred for another 30 min. The liner was placed inside an autoclave (200 mL) and heated in an oven for 10 h at 180 °C. After cooling to RT, the precipitate was separated by centrifugation and washed three times with ethanol. The resulting BMA-2 was dried at 80 °C for 24 h.

### Modification of Solvothermally Prepared BMA-2 by IL-PO_2_

The BMA-2-IL-PO_2_ was prepared similarly to the parental BMA-2 on a smaller scale using a 50 mL autoclave. The ionic liquid IL-PO_2_ (0.1800 g; 1 g of IL-P/5 mmol of Mg^2+^) was stirred together with magnesium nitrate hexahydrate (0.2059 g; 0.8 mmol; 2 equiv), aluminum nitrate nonahydrate (0.1500 g; 0.4 mmol; 1 equiv), isopropyl alcohol (31 mL), and glycerol (6 mL) in a Teflon liner, and subsequently placed into the autoclave and heated in an oven (180 °C, 10 h). After centrifugation and one ethanol washing, the product was dried in an oven and cooled to RT in a vacuum desiccator.

### Microwave-Assisted Ring-Opening Polymerization of εCL

A monomodal microwave reactor (Discover SP Microwave synthesizer, CEM Corporation) operating at a 2450 MHz frequency was used for studying the initiating/catalyzing effect of the modified BMAs on the ROP of εCL. Two types of the BMAs were tested as follows.

#### Solvolytically Prepared BMA (Denoted BMA-1)

Prior to the polymerization, εCL was added under an argon atmosphere into the BMA-1 dispersion and the mixture was subsequently dried under vacuum to eliminate the solvent (methanol, residual water content) present in the dispersion. Then, the mixture was transferred into a sealed 10 mL flask and sonicated in an ultrasonic bath for 10 min.

#### Solvothermally Prepared BMA (BMA-2) and Its IL-Modified Analogue (BMA-2-IL-PO_2_)

Prior to the polymerization, the BMA-2 or BMA-2-IL-PO_2_ powder was dried at 80 °C overnight under vacuum to remove adsorbed water. Then, the powder was homogenized with εCL using a vortex (2000 rpm) and sonicated in an ultrasonic bath for 10 min.

Subsequently, the flask with a mixture of εCL and the modified BMA was placed inside the microwave reactor and heated at a steady power of 30 W with a limited maximum temperature of 170 °C to avoid thermal degradation. During microwave heating, the temperature was consistently tracked with a built-in infrared thermometer. The polymer yield was determined gravimetrically after the extraction of the polycaprolactone (PCL) product with distilled water (three extraction cycles of 20 min each at room temperature). The prepared PCL was characterized using size exclusion chromatography (SEC) and matrix-assisted laser desorption/ionization coupled with time-of-flight mass spectrometry (MALDI-TOF MS).

## Instrumental Part

### Scanning Electron Microscopy (SEM)

An FEI Nova NanoSEM 450 microscope was used for high-resolution scanning electron microscopy (HRSEM) measurements with a through-lens detector (TLD). The measurements were primarily conducted under a high-vacuum environment with an acceleration voltage of 10 kV. BMA-2-IL-PO_2_ was measured in low vacuum (80 Pa) at a 10 kV acceleration voltage. The samples were obtained from very diluted methanol suspensions, which were dropped onto silicon chips and dried in air. The EDS standardless semiquantitative analysis was performed on the same microscope operating at 10 kV, equipped with an Everhart-Thornley detector (ETD), using an Ultim Max 100 SDD detector and AZtecLive software (Oxford Instruments; Abingdon-on-Thames, The United Kingdom). The concentrated samples for EDS analyses were prepared on Be chips.

### Transmission Electron Microscopy (TEM)

High-resolution transmission electron microscopy (HRTEM) was measured using a Talos F200X TEM microscope (FEI, Czech Republic) with an accelerating voltage of 200 kV. Standard copper grids coated with a thin, transparent, holey carbon film suitable for high-resolution TEM observation of the samples were used for sample preparation. The fine methanolic suspension was placed in an ultrasonic bath for a few minutes and then 5 μL was dropped onto a copper grid. The samples were freely dried at room temperature.

### X-ray Powder Diffraction (XRPD)

Diffraction patterns of all products were collected with a PANalytical X́PertPRO MPD diffractometer equipped with a conventional X-ray tube (Cu_Kα_ radiation, 1.5418 Å, 40 kV, 30 mA, line focus) and a linear position-sensitive detector PIXCel^1D^ with an antiscatter shield. X-ray patterns were measured in the range of 5° to 55° 2Θ with a step of 0.0131° and 300 s counting per step producing a scan of about 1 h 20 min. Conventional Bragg–Brentano geometry was used with the following parameters: 0.04 rad Soller slit, 0.5° divergence slit, and 15 mm mask in the incident beam; 0.5° antiscatter slit, 0.04 rad Soller slit, and Ni beta-filter in the diffracted beam. The samples of solvolytic materials were prepared by dropping the alcoholic suspension onto a silicon zero-background holder and subsequently air-drying, resulting in a thin film. The sample of BMA-2 was prepared similarly from a cyclohexane suspension. The BMA-2-IL-PO_2_ sample was prepared with front filling. The background of the spectra was subtracted with the HighScore Plus software package (Malvern PANalytical, The Netherlands, version 5.3.1).^15^

### Fourier Transform Infrared Spectroscopy (FTIR)

The measurements were carried out by a Nicolet Nexus 670 FTIR spectrometer equipped with an ATR interface with a diamond crystal, in the range of 4000 to 400 cm^–1^ (64 scans, resolution 4 cm^–1^). In the case of solvolytically prepared materials, a concentrated suspension of the sample was dropped onto the sampling area, and after evaporation, it was measured. Solvothermally prepared samples were measured as powder directly on the ATR crystal.

### Simultaneous Thermal Analysis Coupled with Mass Spectrometry (STA/MS)

The materials’ thermal stability and characterization were measured using the simultaneous thermal analysis (thermogravimetric analysis (TGA) and differential scanning calorimetry (DSC)) with a Netzsch STA449 F1 Jupiter instrument coupled with mass spectrometry Agilent Technologies 5977B MSD (STA/MS). The 5977B Series MSD is a single-quadrupole GC/MS system with direct MS use. The mass spectrometry was used to detect the *m*/*z* of specific values, carefully selected depending on the studied material. Measurements of the thermal stability and characterization of the materials were performed in an alumina crucible, with the purge gas argon at a flow rate of 50 mL/min. The temperature program for the measurements was 35–750 °C with a heating rate of 5 °C/min, and the mass of the samples was around 3 mg. The instrument was calibrated using indium, zinc, aluminum, and silver. The *m*/*z* values represent water (18), glycerol (29, 31), isopropyl alcohol (29, 46), ethanol (29, 31, and 46), and methanol (31, 32). The *m*/*z* values chosen for IL-PO_2_ were 63 for PO_2_, 79 for PO_3_, 95 for PO_4_, and 129 for C_8_H_17_O.

### X-ray Photoelectron Spectroscopy (XPS)

XPS spectra were obtained using a Phoibos 100 X-ray photoelectron spectrometer (SPECS) operating in fixed analyzer transmission (FAT) mode with a five-channel MCD-5 detector (SPECS). An achromatic X-ray source, XR50 (SPECS), was used with an Al/Mg dual X-ray anode. The spectra were collected using an X-ray Kα line (energy of 1486.6 eV) at 12 kV and 200 W. A flood gun FG 20 (SPECS) was used at 1 eV and 20 μA. The sample was placed on a double-sided carbon conductive adhesive tape on a stainless-steel sample holder. The survey spectrum was acquired at a pass energy of 40 eV, and high-resolution spectra were acquired at a pass energy of 10 eV. The spectra were analyzed in CasaXPS using the Shirley background model and built-in RSF. The bonding energies (BE) reported here are mainly referenced to C–C bonds in the C 1s peak, although it is known not to be a universal solution.^16,17^ But this method yields usually reasonably good results given the experimental uncertainty. There is reported BE referencing with an internal standard, setting Mg 2p to 50 eV,^18^ or a discussion of the possibility of using O 1s instead of the typical C 1s for referencing.^16^

### Size Exclusion Chromatography (SEC)

Size exclusion chromatography (SEC) was used to determine the number-average (*M*_n_) and weight-average (*M*_w_) molar mass, as well as dispersity (*M*_w_/*M*_n_) of PCL. The analyses were performed using a GPC system equipped with a set of three PLgel columns with pore sizes of 50/10E3/10E4 Å, a length of 300 mm, an inner diameter of 7.5 mm, and a particle size of 10 μm (Agilent, Santa Clara, CA, USA) and two detectors: a refractive index detector Shodex RI-101 (Showa Denko, Kawasaki, Japan) and a UV–vis detector LCD 2084 (Ecom, Prague, Czech Republic). THF was used as the mobile phase at a flow rate of 1.0 mL/min. The PCL samples were prepared at a concentration of 5 mg/mL and filtered through a 0.45 μm PTFE membrane (Whatman) before analysis. Calibration was conducted using polystyrene standards.

### Matrix-Assisted Laser Desorption/Ionization Coupled to Time-Of-Flight Mass Spectrometry (MALDI-TOF MS)

MALDI-TOF mass spectra were acquired using the UltrafleXtreme TOF–TOF mass spectrometer (Bruker Daltonics, Bremen, Germany) equipped with a 2000 Hz smartbeam-II laser (355 nm) in the positive ion reflectron mode. Panoramic pulsed ion extraction and external calibration were used for molar weight assignment. The dried droplet method was used in which the solutions of the sample (10 mg/mL), the matrix DHB (2,5-Dihydroxybenzoic acid; Sigma–Aldrich, 98%, 20 mg mL^–1^), and the ionizing agent sodium trifluoroacetate (CF_3_COONa; Sigma–Aldrich, 10 mg mL^–1^) in THF (Sigma-Aldrich, anhydrous, 99.9%) were mixed in a volume ratio of 4:20:1. One microliters of the mixture was deposited on a ground steel target.

## Results and Discussion

As mentioned above, two synthetic pathways were chosen to obtain bimetallic alkoxides containing Mg^2+^ and Al^3+^ cations. The solvolytic method was chosen to achieve a very low amount of water content^7^ using volatile solvents (methanol and ethanol). The solvothermal method was selected to receive more stable alkoxides using the nonvolatile solvent (glycerol). The pristine BMA-1 and BMA-2 were modified with IL-PO_2_, which was selected for its previously proven catalytic ability in ROP.^2,4,19^ As the solvolytically prepared BMAs were reported as layered materials,^7^ we decided on a one-pot modification of pristine BMA, which is an effective method of IL intercalation into layered materials.^4^ We hypothesized that the expected layered BMA particles with intercalated IL could be an effective catalyst for ROP. The second solvothermal method was selected because the resulting BMA was expected to be more stable, which would be practical from the perspective of the catalytic application.

### Solvolytically Prepared BMAs

BMA-1 is a white powder without a preferred shape, forming aggregates of approximately 1 μm or larger and containing pores (Figure 1a), as can also be better seen in the separated particles shown in the TEM image (Figure 1b). The pores could have been formed due to the evaporation of alcohols. SEM images of the BMA-1-IL-PO_2_ form very similar aggregates compared to the pristine BMA-1 (Figure 1c). As can be seen in the image Figure S1 for BMA-1-IL-PO_2_, some other phase is present in the form of a fiber.

**Figure 1:**
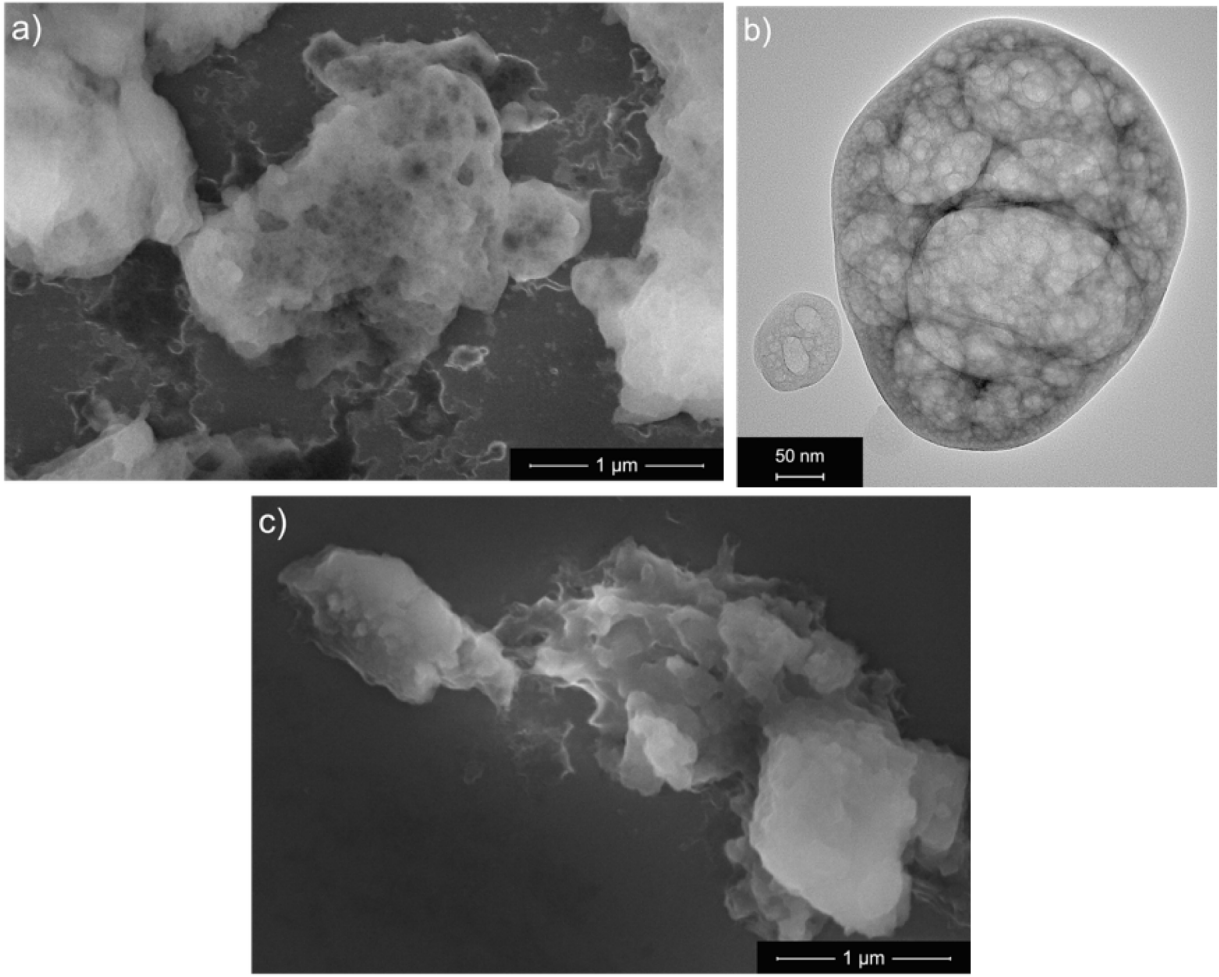
a) SEM and b) TEM images of BMA-1; c) SEM image of BMA-1-IL-PO_2_.

The EDS analysis images of BMA-1 (Figure 2) and BMA-1-IL-PO_2_ (Figure S2) reveal the presence of the expected elements (Mg, Al, C, O) distributed throughout the particles. BMA-1 contains residual Na from the starting material in less than 0.5 at.%. BMA-1-IL-PO_2_ contains around 4 at.% of Na (Figure S2, Table S1), which may explain the presence of a second, morphologically different, sodium-rich phase in Figure S1. Further washing of BMA-1-IL-PO_2_ with methanol, as was done for BMA-1, led not only to the elimination of the sodium-rich species but also to the washing out of IL-PO_2_. Hence, the one-pot synthesis was probably not suitable or effective for IL-PO_2_ modification of BMA-1, resulting in weaker surface grafting.

**Figure 2:**
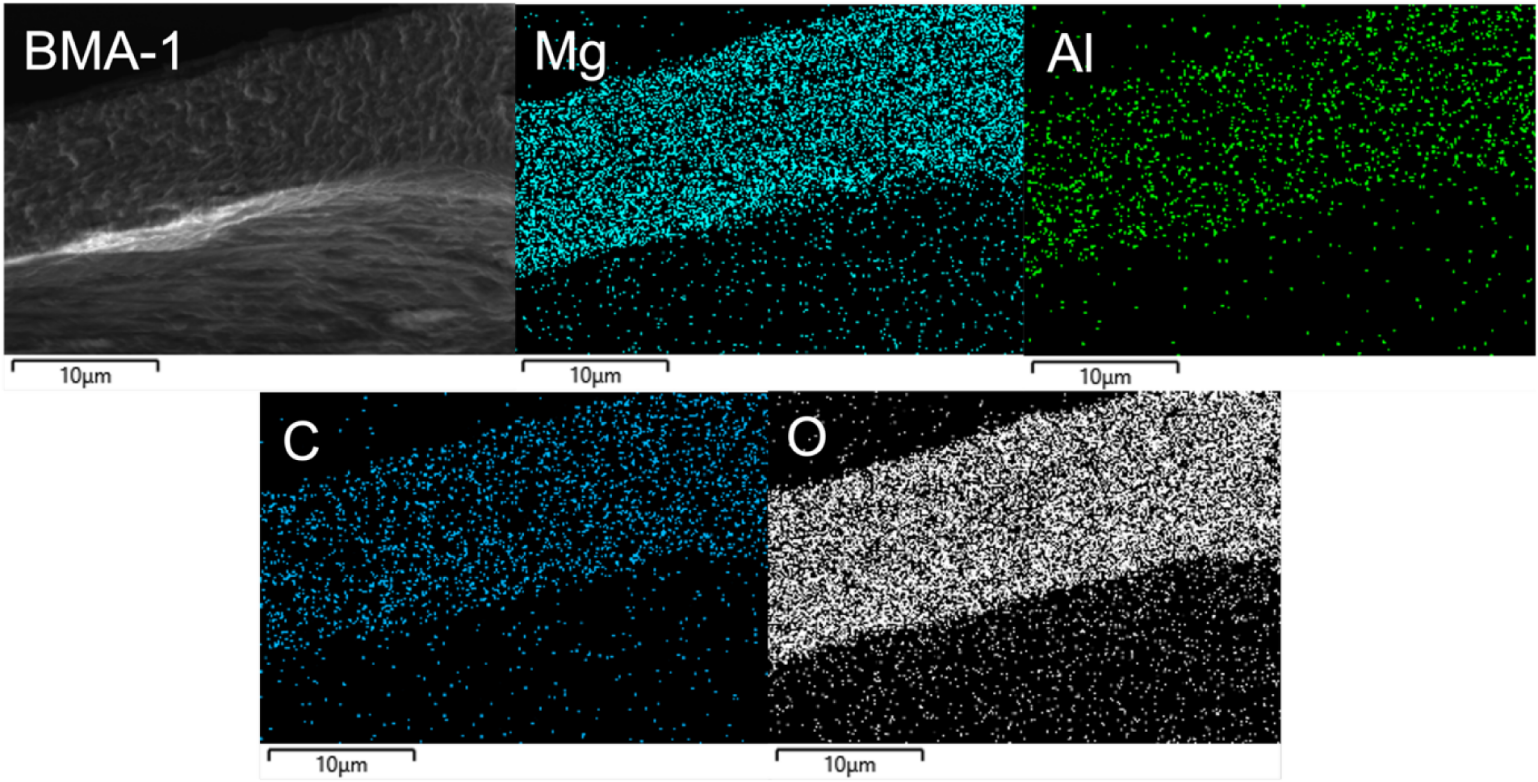
EDS analysis images of BMA-1. Mapping and spectra are shown.

Although the ratio of Mg^2+^ and Al^3+^ reactants was set to 2:1, the resulting materials evinced a ratio of 5.5:1 for BMA-1 and 3.4:1 for BMA-1-IL-PO_2_ (based on data in Table S1). This shift toward Mg might be explained by the different pH in the respective reaction mixtures caused by the presence of IL-PO_2_. As for LDH, the higher the pH of the mixture, the greater the amount of Mg that the product contains.^20^

The XPS analysis, like the EDS analysis, also confirmed the presence of the expected elements Al, Mg, O, and C in all four samples. Additionally, BMA-1 and BMA-1-IL-PO_2_ also contained Na from the preparation and P (only for the modified sample). The compositions are summarized in Table 1. The Ar gas used was detected in the spectra of all samples but was excluded from the calculations.

**Table 1:** Surface Composition from XPS High-Resolution Spectra

Sample	Al [at.%]	C [at.%]	Mg [at.%]	Na [at.%]	O [at.%]	P [at.%]
BMA-1	2.9	21.8	22.2	1.6	51.5	0
BMA-1-IL-PO_2_	2.2	44.6	9.3	7.3	34.4	2.2

The detailed analyses of high-resolution XPS spectra show the differences between the preparation routes (Figures 3, 9, S8). The binding energies (BEs) of selected components are summarized in Table 2. The related literature on similar materials reports that BEs vary for Al 2p from 73.88 eV up to 75.1 eV for aluminum^21–24^ and for Mg 2p from 50 eV up to 51 eV.^18,24^ All BEs are reported as oxide/hydroxide structures.^25^

**Table 2:** Binding Energies from XPS for Selected Elements and Auger Parameter for Mg

Sample	Al 2p [eV]	Mg 2p [eV]	Mg KLL [eV]	Na 1s [eV]	P 2p [eV]	O 1s [eV]	C 1s C–C [eV]	Mg α [eV]
BMA-1	74.6	50.2	1179.7	1071.8	-	531.8	284.8	1229.9
BMA-1-IL-PO_2_	74.8	50.1	1180.1	1071.6	132.8	531.6	283.7	1230.2

**Figure 3:**
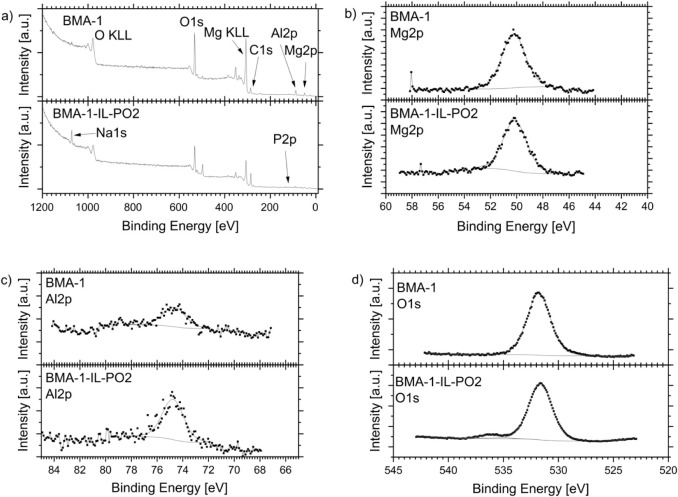
XPS: a) Survey spectra and high resolution spectra of b) Mg 2p, c) Al 2p, and d) O 1s for BMA-1 and BMA-1-IL-PO2.

The Mg bonding structure can be studied using the Auger parameter α.^16^ The Auger parameters for all measured samples are approximately 1230 eV, as shown in Table 2. This value is closer to the 1230.5 eV reported for Mg­(OH)_2_ than to the 1231.3 eV reported for MgO. The BE of Mg 2p, about 50.4 eV, reported here also supports the presence of Mg­(OH)_2_, where the literature reports the BE of Mg 2p at 49.8 eV, and for MgO, it reports Mg 2p at 49.4 eV with identical referencing to C 1s.^16^

The carbon-related peak C 1s is reported in the literature, e.g., AlMg-LDH/biomass carbon fiber,^23^ with a C–C bond at an unusually low BE of about 283.2 eV, with the main component C–O at 284.8 eV. Because of the high content of oxygen in the samples (up to 50 at. %), the most intense peak in C 1s is probably due to C–O bonding. Then, the C–C bond was measured at BE 284.8 eV for BMA-1 as the most common value except for the sample BMA-1-IL-PO_2_ where the C–C bonds must be identified at a low 283.7 eV, similar to some literature.^23^ Although the BE of other elements is very similar between samples, see Table 2, Mg 2p was used in this sample as an internal standard.^18^

The oxygen-related O 1s peaks have mainly a single peak character, with the exception of BMA-1-IL-PO_2_, where an additional small contribution is observed at the higher energy of 536.2 eV. The sample BMA-1-IL-PO_2_ has a higher content of sodium, and such an unusually high BE observed in the O 1s peaks is not caused by photoelectrons belonging to O 1s photoelectrons; instead, they are caused by the presence of Auger electrons related to the presence of Na in the sample.^27^ Such features were observed, for example, on nanosized NaO_2_ particles and generally on sodium-rich surfaces and electrolytes.^26–28^ It is probable that Na here is an oxide or hydroxide as indicated by FTIR and DSC. The P 2p peak at 132.8 eV is symmetrical, and no increase in background was observed for P 2p photoelectrons; thus, these photoelectrons do not penetrate an overlayer, and we can expect the P to be distributed mainly on the most top surface. The hexametaphosphates in LDH structures are reported at P 2p about 134 eV, but the Al 2p and Mg 2p peaks are detected at higher binding energies than those reported here.^29^ Therefore, the formation of metallic phosphinate salts is ruled out. In the case where IL-PO_2_ was present as a lubricant additive to a polyalphaolefin base oil, a P 2p doublet was observed at 134.0 and 135.0 eV, evidencing a mixture of several PO_2_-based additives.^30^

The XRD patterns of solvolytically prepared BMAs before and after modification with IL-PO_2_ are shown in Figure 4a. Two broad diffraction lines are observed for both solvolytically prepared samples at approximately 9.4° and 18.6° 2Θ, which agree with those of similar BMAs prepared previously.^7,12^ The crystallinity of all samples is not very high, and the intensity of the diffraction lines for the BMA-1-IL-PO_2_ decreased with a negligible shift, indicating that IL-PO_2_ was not intercalated but rather grafted onto the BMA-1 surface. This finding is in agreement with the results of XPS and EDS (see Figure S2).

**Figure 4:**
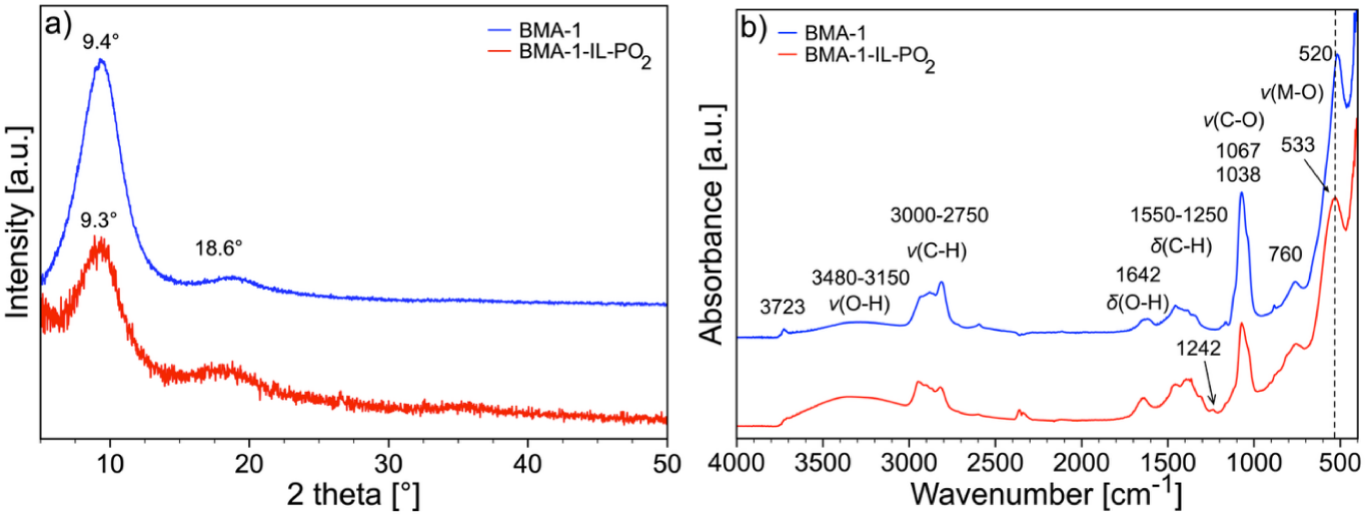
a) XRPD patterns and b) FTIR spectra of solvolytically prepared BMA-1 and BMA-1-IL-PO_2_.

The FTIR spectra of both solvolytically prepared BMA samples are shown in Figure 4b. The low-intensity broad bands at 3480–3150 and 1642 cm^–1^ were attributed to the stretching and deformation vibrations of O–H groups, indicating small amounts of water and/or residual alcohols in the samples. Additionally, the weak band at 3723 cm^–1^ was assigned to the O–H stretching of non-hydrogen-bonded OH moieties.^31^ The absorption bands between 2750 and 3000 cm^–1^ and 1550–1250 cm^–1^ were assigned to the stretching and bending vibrations, respectively, of C–H bonds in alkoxides (methoxides or ethoxides). The intense bands at 1067 and 1038 cm^–1^ correspond to the C–O stretching vibrations of alkoxide (methoxide or ethoxide) and/or residual alcohol (methanol or ethanol), respectively.^32,33^ In addition, the bands at 760 and 520 cm^–1^ were assigned to M–O (M = Mg, Al) stretching vibrations.^33,34^ The immobilization of the IL-PO_2_ on the BMA-1 surface was seen by specific modes as the P=O vibrations at 1242 cm^–1^.^19^ The shift of the M–O vibration from 520 to 533 cm^–1^ observed for BMA-1-IL-PO_2_ could be explained by the interactions between alkoxides and IL-PO_2_.

The thermal behavior of the prepared BMAs was also studied using TGA/MS (see Figure 5), with the primary objective of verifying whether the prepared BMAs can be applied as catalysts in polymerizations requiring sufficient thermal stability (<150 °C) and low water content. While the first step of thermal decomposition corresponded to the release of free water, the second step represented the total decomposition of the material, which was accompanied by the evaporation of water (*m*/*z* 18) and alcohols present in the material—methanol (*m*/*z* 32), ethanol (*m*/*z* 46), and a partial group arising from their decomposition (*m*/*z* 31). BMA-1 appears to be a stable material; the main temperature onset (370 °C) corresponds to decomposition. These temperatures of thermal decomposition corresponded to those of the hydroxides, namely aluminum and magnesium hydroxides.^35,36^ Also, from the DSC records (see Figure S6), it is evident that Mg^2+^ and Al^3+^ hydroxides were present in the samples.^37,38^ BMA-1, as studied by TGA/MS, showed a mass loss of 40–50%. At the beginning, the water content of BMA-1 as free water is approximately 6 wt %, and, as MS records show, the bonded water is around 4.8 wt %. Interestingly, the record for BMA-1 shows just a small amount of ethanol present in the sample. During the reaction, the more acidic and more abundant methanol shifted the alcohol equilibrium toward methoxide.^39^

**Figure 5:**
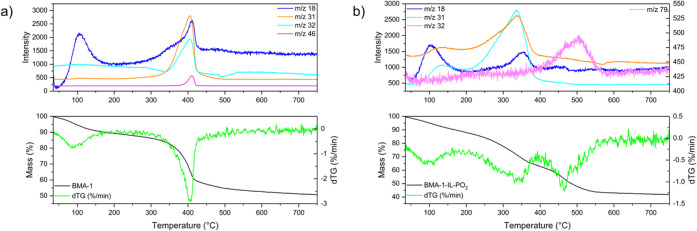
TGA results showing mass loss, derivative thermogravimetric curve (dTG), and the evolution of selected *m*/*z* values as a function of temperature for a) BMA-1 and b) BMA-1-IL-PO_2_. The *m*/*z* values represent water (18), alcohols (methanol: 31 and 32; ethanol: 46), and IL-PO_2_ (79 for PO_3_).

The TG results reveal the presence of free (∼8 wt %) and structure-bonded (∼5 wt %) water in BMA-1-IL-PO_2_. While the TG record of BMA-1 showed only one main degradation step, the decomposition of BMA-1-IL-PO_2_ consists of two steps, where the onset temperatures are approximately 275 and 443 °C (see Figure 5). Compared with the behavior of unmodified BMA-1 and IL-PO_2_ (Figures 5a and S5), it can be concluded that the modification of BMA-1 was successful. Also, the mass loss of BMA-1 relative to BMA-1-IL-PO_2_ clearly shows the difference. While the BMA-1 loss is approximately 50 wt %, the BMA-1-IL-PO_2_ loss is approximately 60 wt % of the starting mass. This means that the presence of IL-PO_2_ is around 10 wt %.

### Solvothermally Prepared BMAs

To the best of our knowledge, no alkoxides with magnesium and aluminum have been published so far by the solvothermal method. Herein, the solvothermal method produced BMA-2 as spherical particles with a diameter of approximately 1 μm and a relatively smooth surface (see Figure 6a). In contrast, BMA-2-IL-PO_2_ exhibited a rough surface and diameters ranging from tenths to units of micrometers (Figure 6b). The roughness of the spherical particles is obviously related to the metal composition. e.g., the study of Wang et al. introduced a set of FeNi glycerolates which form microspherical particles (diameter ca. 1 μm) with a rough surface.^13^ In contrast, heterometallic FeNiCoCrMn glycerolate creates smooth-surfaced microspheres of similar size.^40^

**Figure 6:**
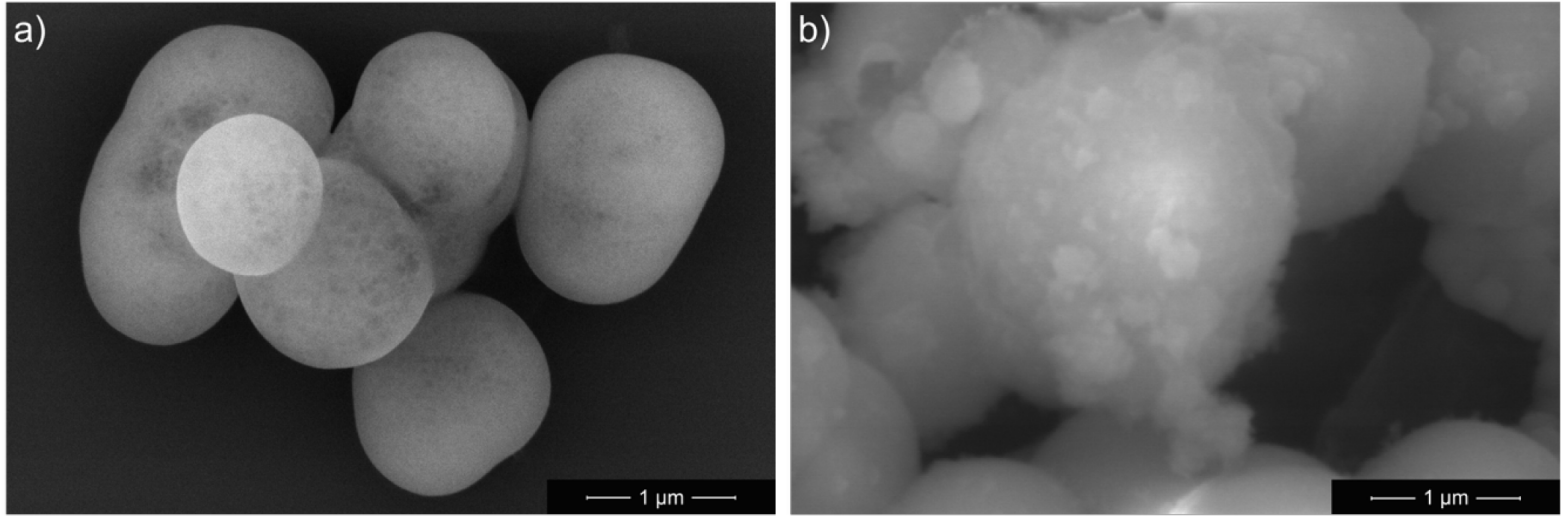
SEM images: a) BMA-2 and b) BMA-2-IL-PO_2_.

The EDS analysis images (Figure 7) for the confirmed expected elements (Mg, Al, C, and O) cover the particles seen in the SEM image. The BMA-2-IL-PO_2_ also contains phosphorus (Figure 8), which is grafted onto the BMA-2 particles; no other contamination was seen. Table S1 shows the SEM-EDS analysis results and documents the trend in the Mg:Al ratio, which has been already described for solvolytic BMAs.

**Figure 7:**
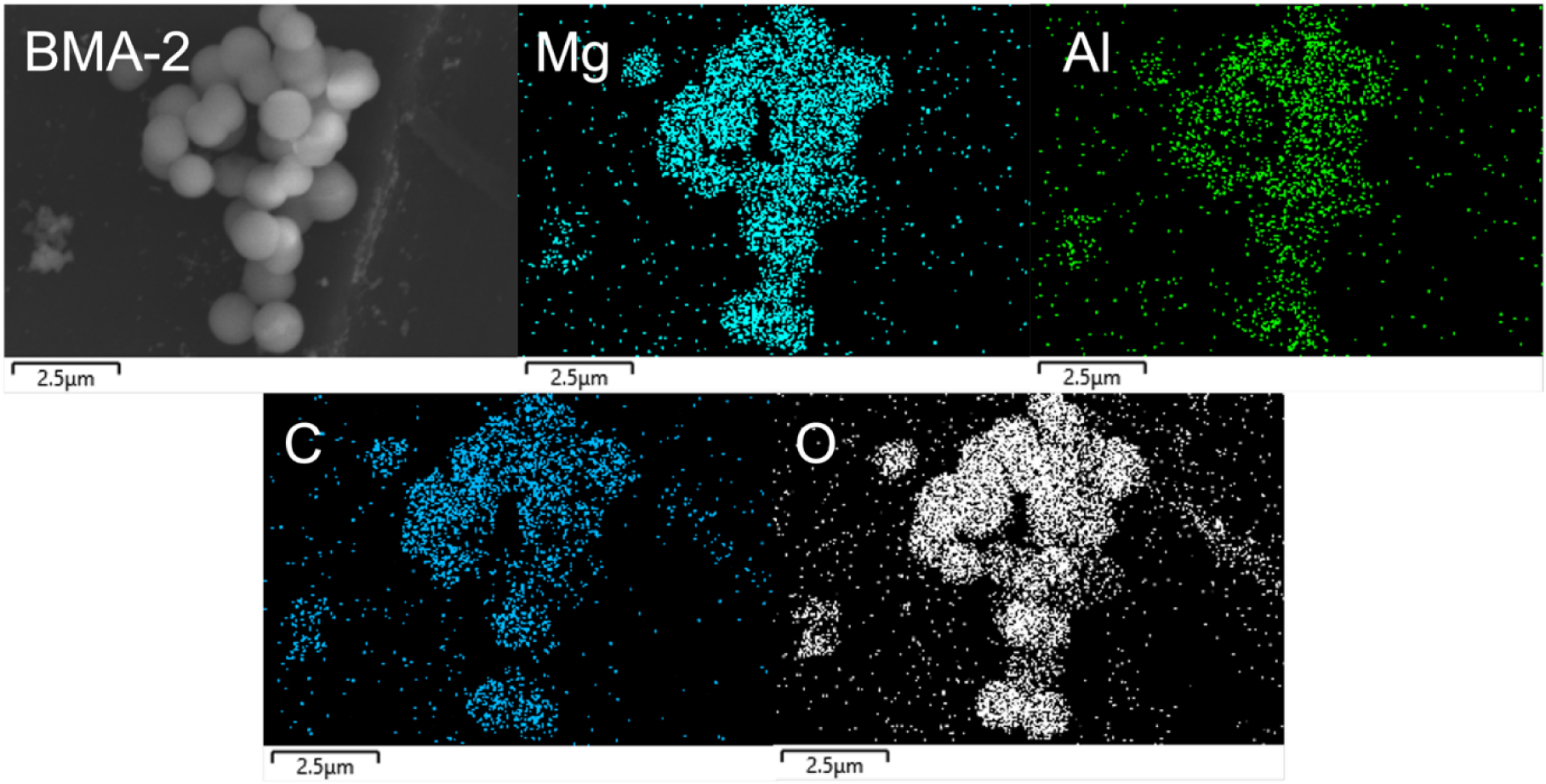
EDS analysis images of BMA-2. Mapping and spectra are shown.

**Figure 8:**
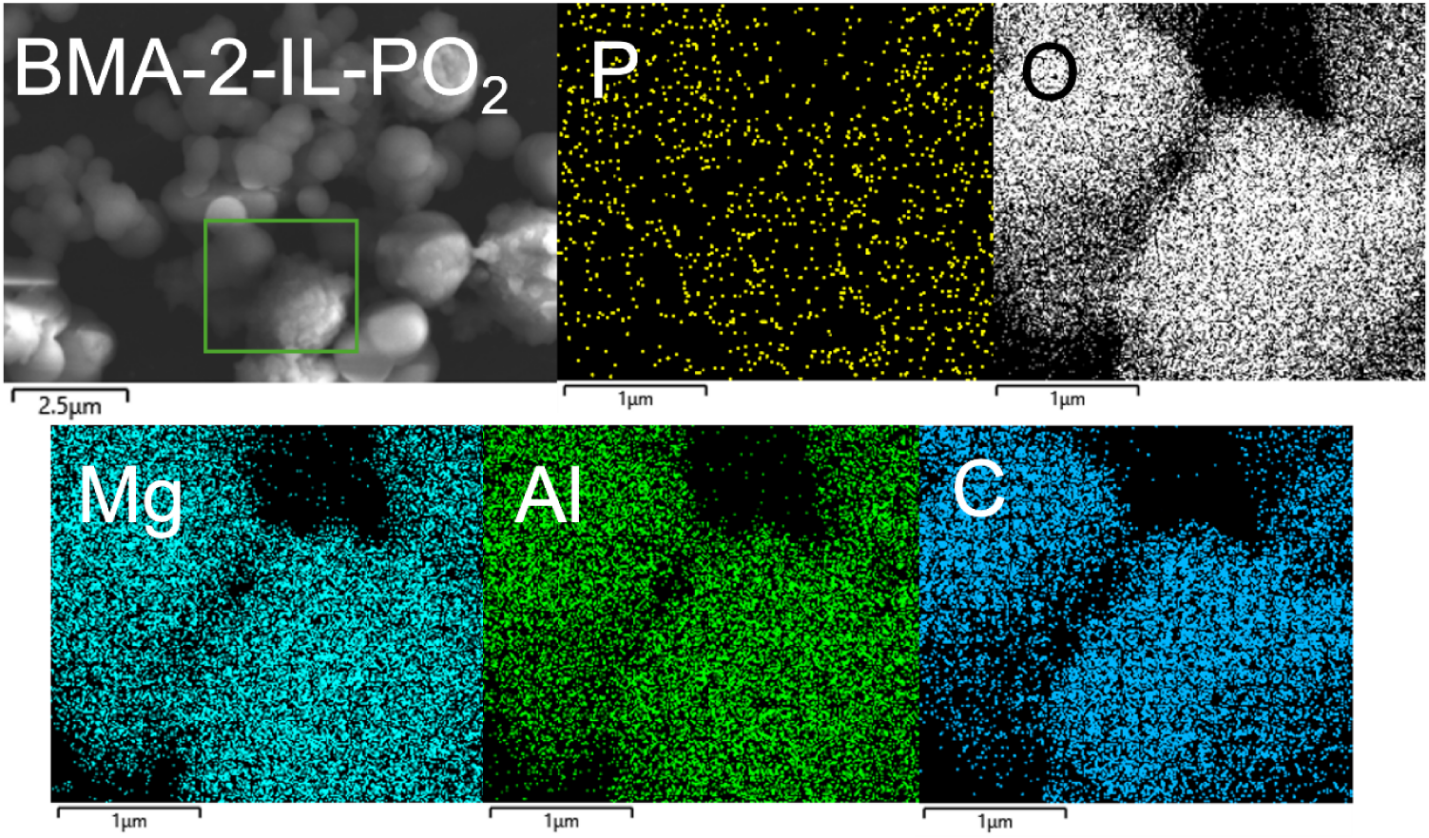
EDS analysis images of BMA-2-IL-PO_2_. Mapping and spectra are shown.

The XPS analysis, as was already mentioned above for BMA-1-based samples, confirms the presence of the expected elements Al, Mg, O, and C in all samples and phosphorus for BMA-2-IL-PO_2_. The compositions are listed in Table 3.

**Table 3:** Surface Composition from XPS High-Resolution Spectra

Sample	Al [at.%]	C [at.%]	Mg [at.%]	Na [at.%]	O [at.%]	P [at.%]
BMA-2	2.8	43.5	12.5	0	41.3	0
BMA-2-IL-PO_2_	4.1	46.8	8.1	0	40.2	0.8

The detailed analyses of high-resolution XPS spectra are shown in Figures 9 and S8. The BE of the selected components is summarized in Table 4. As mentioned, for solvolytic samples, the results for solvothermal correspond more to those of hydroxide. The Auger parameters for BMA-2 and BMA-2-IL-PO_2_ samples are closer to the value of Mg­(OH)_2_ (1230.5 eV) than to that of MgO (1231.3 eV).^16^ The BE of Mg 2p is at 50.7 eV for BMA-2 and at 50.6 eV for BMA-2-IL-PO_2_, which is closer to the value for Mg­(OH)_2_ (49.8 eV) than for MgO (49.4 eV).^16^

**Table 4:** Binding Energies from XPS for Selected Elements and Auger Parameter for Mg

Sample	Al 2p [eV]	Mg 2p [eV]	Mg KLL [eV]	Na 1s [eV]	P 2p [eV]	O 1s [eV]	C 1s C–C [eV]	Mg α [eV]
BMA-2	74.8	50.7	1179.6	-	-	532.3	284.8	1230.3
BMA-2-IL-PO_2_	74.6	50.6	1179.7	-	133.1	532.2	284.8	1230.3

**Figure 9:**
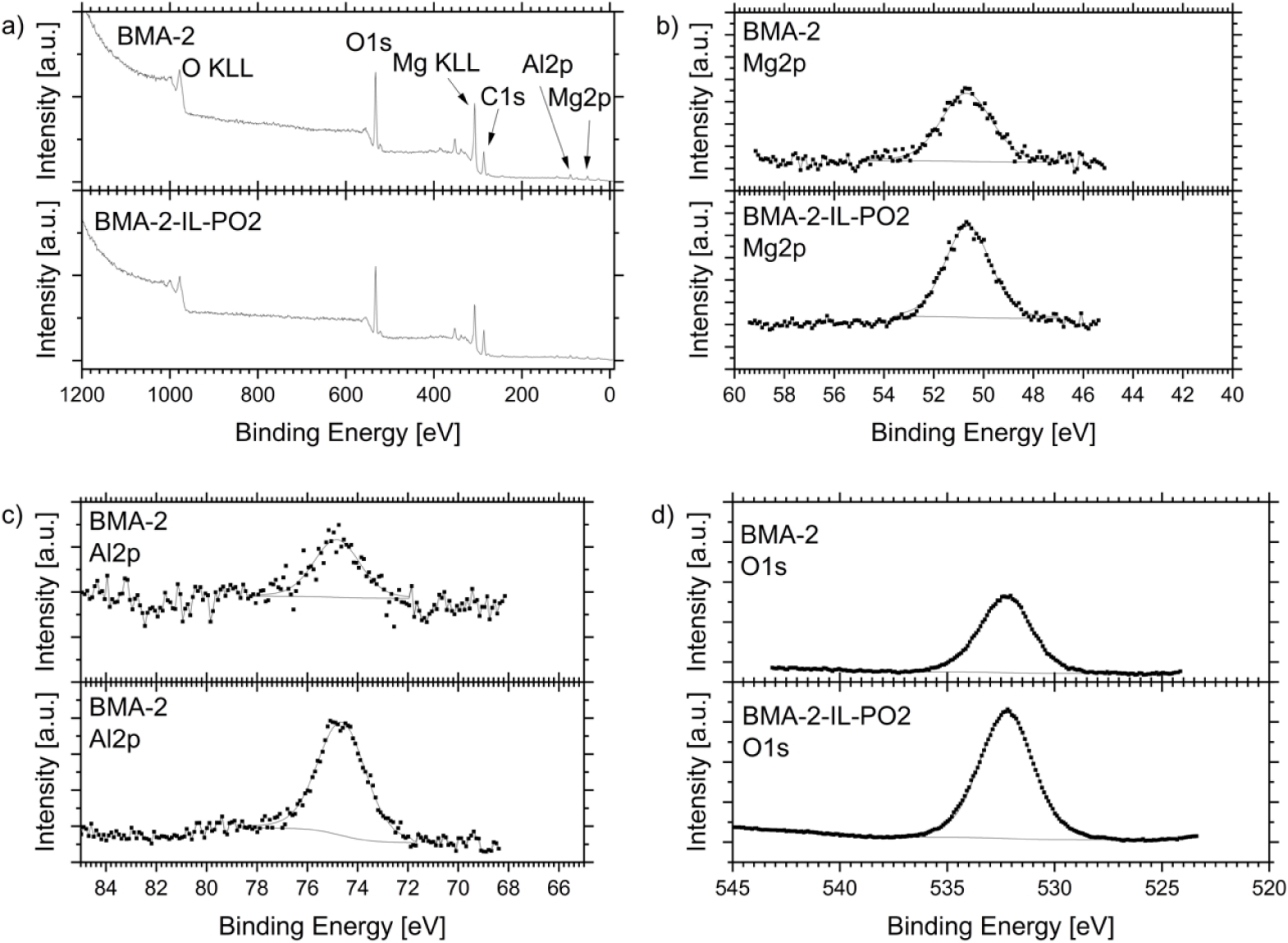
XPS: a) Survey spectra and high resolution spectra of b) Mg 2p, c) Al 2p, and d) O 1s for BMA-2 and BMA-2-IL-PO2.

The O 1s main peak for BMA-2 samples is systematically about 0.5 eV higher than that for BMA-1 samples, and a similar shift can be observed on Mg 2p, although the Auger parameter is not changed. Also, phosphorus added as PO_2_ shows an identical 0.4 eV shift between the different preparation routes to 133.1 eV (see Figure S8). BMA-2-IL-PO_2_ has some increase in background for the P 2p photoelectron; thus, those photoelectrons should partially lose he energy in some overlayer. Therefore, phosphorus might be incorporated deeper into the structures within the top 10 nm of the surface.^41^ Because the difference in the background is observed on the P 2p, the other elements should be distributed similarly to the BMA-1 samples. However, due to the different location of phosphorus in the structure, we can expect changes in the surroundings and, therefore, the above-discussed changes in BE.

The XRD pattern of BMA-2 shows broad diffraction lines at 8.2° and 21.6° 2Θ, and with very low intensity at 37.1° 2Θ (Figure 10a). The diffraction line at 8.2° 2Θ is shifted to a smaller angle than in the solvolytically prepared BMA-1, due to the sterically more demanding glycerol molecule. BMA-2-IL-PO_2_ exhibits a higher intensity of diffraction lines and displays a shift from 8.2° to 9.9° 2Θ, which could be attributed to modification by IL-PO_2_.

**Figure 10:**
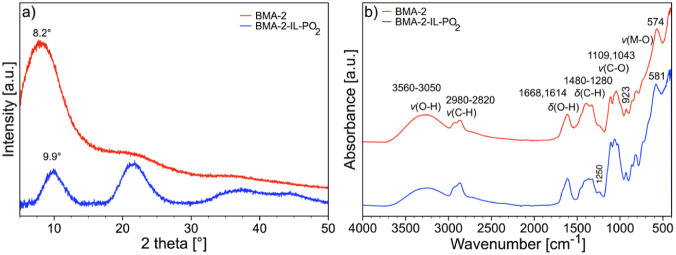
a) XRPD patterns and b) FTIR spectra of solvothermally prepared BMA-2 and BMA-2-IL-PO_2_.

The FTIR spectrum of BMA-2 (Figure 10b) shows the presence of an O–H bond with higher intensities of stretching (3560–3050 cm^–1^) and deformation (1668, 1614 cm^–1^) vibration than for the solvolytically prepared BMAs, indicating the presence of OH groups from glycerol and/or water (presented already from starting materials). The vibration modes for glycerolate are found at 2980–2820 cm^–1^ ν­(C–H), 1480–1280 cm^–1^ δ­(C–H), and 1109, 1043, and 923 cm^–1^ ν­(C–O).^42^ The band at 574 cm^–1^ is attributed to M–O (M = Mg, Al) stretching vibrations.^33,34^ The FTIR spectrum of BMA-2-IL-PO_2_ (Figure 10b) shows a vibration mode of the P=O band at 1250 cm^–1^ and the C–H bonds overlap with the C–H vibrations of glycerol in the region of 2850–2960 cm^–1^.^19^ The M–O vibration slightly shifted from 574 to 581 cm^–1^, which could be explained by interaction with IL-PO_2_.

TGA of the BMA-2 showed the first weight loss at 90–150 °C due to the release of surface-adsorbed water (ca. 3 wt %, *m*/*z* 18, Figure 11a). The second weight loss around 430 °C was connected to the decomposition of BMA-2. At this step, the observed release of *m*/*z* 29 and 31 corresponded to glycerol, isopropanol, and ethanol fragments, while the *m*/*z* 46 signal representing isopropanol and ethanol fragments was absent. This proved the successful formation of BMA-2 containing glycerol. The *m*/*z* 18 fragment released at this step also showed that water (∼7 wt %) was structure-bonded into BMA-2. The overall mass loss of the material at 750 °C was around 57 wt %. TGA of BMA-2-IL-PO_2_ showed the first weight loss below 150 °C, attributed to water release, indicating the presence of approximately 4 wt % of adsorbed water (see Figure 11b). A moderate weight decrease in the temperature range of approximately 230–350 °C was associated with a gradual release of BMA-bonded glycerol, as indicated by the evolution of *m*/*z* 31 ion. Also, the structure-bonded water was reported, with an amount of around 5 wt %. The main thermal decomposition of BMA-2-IL-PO_2_ occurred around 407 °C. During this degradation step, *m*/*z* ion 79 was released, indicating the presence of IL-PO_2_. In the main degradation step, structure-bonded water was also present (∼8 wt %). The overall mass loss of BMA-2-IL-PO_2_ at 750 °C was around 60 wt %. While measurements of BMA-1 clearly showed the difference in mass loss between unmodified and modified samples, BMA-2 exhibited only approximately a 3 wt % difference. As with the BMA-1 samples, the DSC records for the BMA-2 samples show evidence of the presence of Mg^2+^ and Al^3+^ hydroxides (see Figure S7).^37,38^

**Figure 11:**
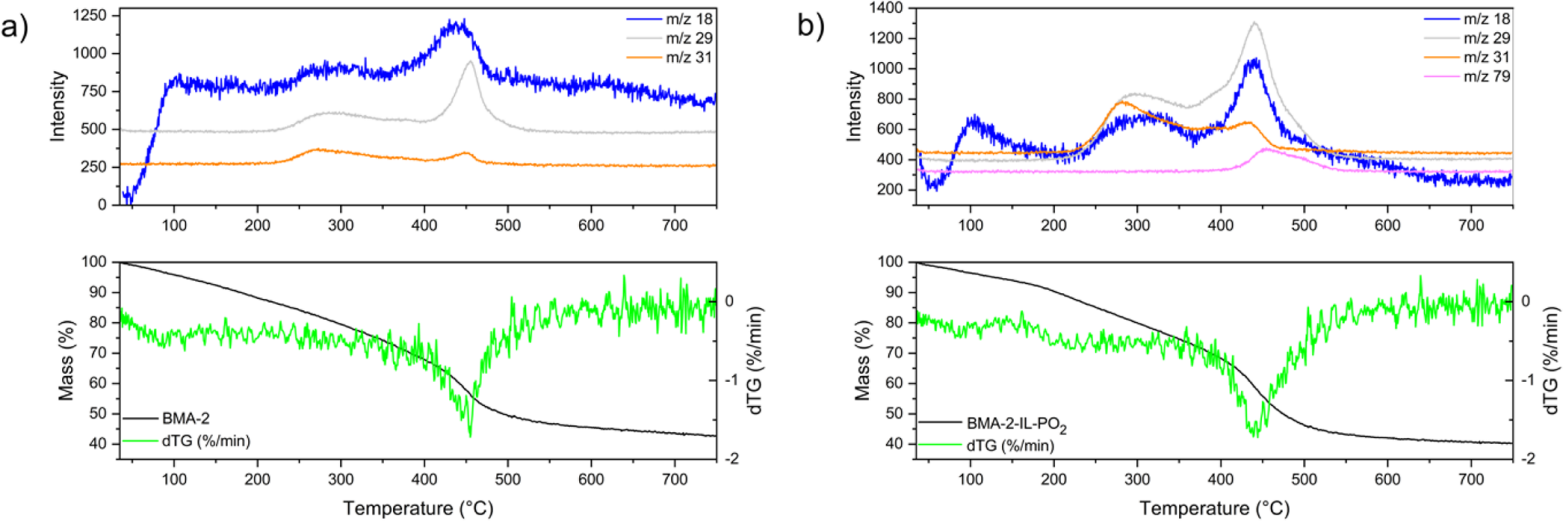
Record of TG, derivative thermogravimetry (dTG), and the chosen values of *m*/*z* for a) BMA-2 and b) BMA-2-IL-PO_2_. The *m*/*z* values represent water (18), alcohols (glycerol: 29, 31), and IL-PO_2_ (79 for PO_3_).

### Ring-Opening Polymerization of ε-Caprolactone under Microwave Irradiation

The ROP of εCL in the presence of pristine BMA and BMA modified with IL-PO_2_ was carried out under microwave irradiation due to the high ability of the εCL monomer and ILs to absorb microwaves, which accelerates the ROP compared to conventional heating methods.^19,43^ The morphological and structural characterizations of BMA-1-IL-PO_2_ show that it additionally contains residual starting material sodium ethoxide (confirmed by SEM and EDS image analysis). Although the impurity is present in small quantities, it is sufficient to influence the ROP mechanism, and therefore should be excluded from the following experiments.

The solvolytically prepared BMA-1 was tested as a catalyst (0.50 wt %) for the microwave ROP of εCL (entry 1 in Table 5). A one-hour reaction at a constant power of 30 W led to a high polymer yield (over 99%). However, only low-molar mass PCL was formed, probably due to the high water content in BMA-1 initiating the growth of polymer chains.^2^ A similar effect was previously reported for LDH particles applied for ROP catalysis.^19^

**Table 5:** Microwave ROP of εCL in the Presence of the Solvolytically Prepared BMA-1, the Solvothermally Prepared BMA-2, and the Modified BMA-2-IL-PO_2_ after 1 h of Reaction at 30 W

Entry	BMA type	BMA content (wt %)	Yield (wt %)	*M*_w_ (g/mol)	*M*_n_ (g/mol)	*M*_w_/*M*_n_
REF^a^	-	0	0	-	-	-
1	BMA-1	0.50	>99	4900	2900	1.69
2	BMA-2	0.50	14	1 400	700	2.00
3	BMA-2	1.00	97	19800	9300	2.13
4	BMA-2-IL-PO_2_	0.25	82	31600	16200	1.95
5	BMA-2-IL-PO_2_	0.50	>99	73700	28900	2.55
6	BMA-2-IL-PO_2_	1.00	98	32600	14900	2.19
7	BMA-2-IL-PO_2_	2.50	99	25200	12000	2.10
8	BMA-2-IL-PO_2_	5.00	>99	18300	7500	2.44
9	BMA-2-IL-PO_2_	10.00	>99	10900	5900	1.85

a Reference run—εCL alone under microwave irradiation.

A slightly different situation occurred in the ROP of εCL in the presence of solvothermally prepared BMA-2 (Table 5). The polymerization in the presence of pristine BMA-2 proceeded relatively slowly and with a low polymer yield (entry 2 in Table 5). The PCL yield increased with a higher addition of BMA-2 (entry 3 in Table 5).

The presence of modified BMA (BMA-2-IL-PO_2_) accelerated polymerization and produced PCL with a higher degree of polymerization (entries 4–9 in Table 5). The molar mass of PCL was influenced by the content of BMA-2-IL-PO_2_ in the reaction mixture. A low content of BMA (0.25 wt %, entry 4) led to a low polymer yield. A content of 0.5 wt % BMA-2-IL-PO_2_ (entry 5) was found to be optimal, since higher contents of BMA (1–10 wt %, entries 6–9) led to a decrease in the average molar mass of the produced PCL. This gradual decrease in *M*_n_ was caused by a high content of initiation centers of BMA promoting the formation of a large number of short PCL chains.

The MALDI-TOF mass spectrometry was used to identify the end groups and to elucidate the initiation mechanism for the solvothermally prepared BMA-2-IL-PO_2_. The highest BMA-2-IL-PO_2_ content was needed for the MALDI-TOF mass spectrometry to receive macromolecules with sufficiently low *M*_n_.^44^ Figure 12 shows the MALDI-TOF mass spectrum of PCL prepared with the BMA-2-IL-PO_2_ (10 wt %) initiating system.

**Figure 12:**
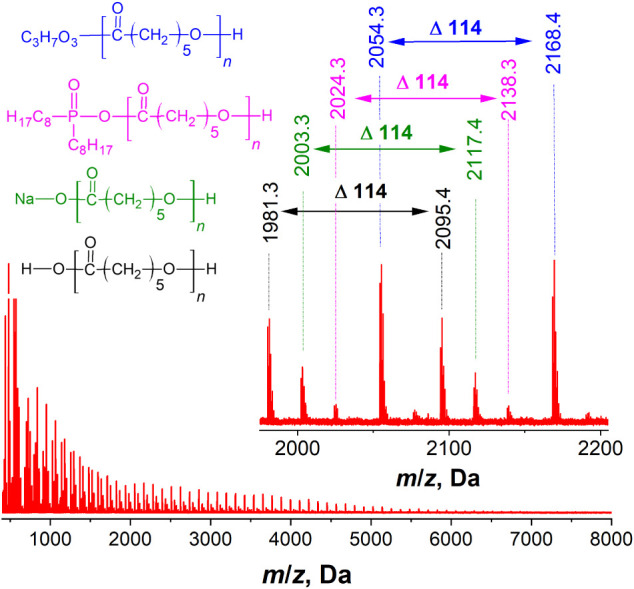
MALDI-TOF mass spectrum of ε-caprolactone/BMA-2-IL-PO_2_ (10 wt %) reaction mixture after 1 h of microwave heating at 30 W. The signals correspond to PCL adducts with Na^+^. The green-marked distribution is formed during MALDI-TOF measurement due to the ion exchange of H^+^ with Na^+^ from the added ionizing agent.

Four distributions of linear PCL chains with repeating units of 114 g/mol were observed. The formation of PCL chains bearing −H and glycerolate end groups (the blue-marked distribution in Figure 12) was observed, providing evidence of the initiation of the alkoxide structure of solvothermally prepared BMA. Additionally, one PCL population of IL-PO_2_-containing species was observed (the magenta-marked distribution in Figure 12), proving that the IL-PO_2_ anion initiated ROP of εCL. The ability of phosphinate anions of ILs to initiate anionic ROP was previously demonstrated by Nguyen et al.^45^ The third and fourth series of signals (the black- and green-marked distributions in Figure 12) represented PCL chains with −H and −OH terminal groups, revealing that the ROP was also partly initiated by traces of water present in BMA-2-IL-PO_2_. Similar to previously published work studying ROP in the presence of LDH, IL-modified particles appear to efficiently catalyze the water-initiated ROP of lactones.^2^

The results thus show that the solvothermally prepared IL-P-modified BMA both initiates and catalyzes the ROP of εCL. As a result, the high molar mass PCL (in the *M*_w_ range of approximately 20–74 kg/mol, Table 5) was produced. Herein, the microwave irradiation activated IL-PO_2_ and water molecules, which became accessible catalytic and initiating sites for the ROP of the monomer molecules of εCL. The progress of ROP in the presence of IL-modified BMA was slower compared to the previously reported polymerizations with IL-modified LDH,^4^ probably due to the slightly lower microwave absorption ability (polarity) of BMA compared to LDH. Compared with LDH, BMA exhibits lower surface polarity due to the replacement of hydroxyl groups by alkoxide moieties and their lower water content. Consequently, the alkoxide materials are less hydrophilic and possess a more organophilic character, which may facilitate the interaction with organic monomers during the ROP of εCL.

## Conclusion

Solvolytic and solvothermal methods were used to prepare pristine and IL-PO_2_-modified BMA via one-pot synthesis. The methods were designed to produce either low-water-content catalysts (solvolytic BMA-1) or more stable materials (solvothermal BMA-2). BMA-1-IL-PO_2_ contained sodium oxide impurities and was therefore excluded from the ROP of εCL.

BMA-1 particles were porous, irregular, and broadly distributed in size, whereas BMA-2 formed spherical particles 1 μm in diameter. After IL-PO_2_ modification, BMA-2-IL-PO_2_ particles increased in size and developed a rougher surface, confirming IL immobilization. Differences in FTIR were observed in the region of M–O and C–O vibrations, likely due to the interaction of IL-PO_2_ with BMAs. The BMA-2 and BMA-2-IL-PO_2_ exhibited an increase in O–H vibrations in the region 3560–3050 cm^–1^, consistent with TGA/MS, indicating slow water removal. The BMA-1 and BMA-1-IL-PO_2_ samples contained free water content of 6 to 8 wt %, while structure-bonded water contents were 4.8 to 5 wt %, respectively. BMA-2 and BMA-2-IL-PO_2_ showed higher structure-bonded water contents (7–8 wt %) and lower free water contents (3–4 wt %). Glycerol has a high affinity for water, and therefore, more water was structure-bonded in the BMA-2 samples. All samples were thermally stable up to 170 °C, sufficient for ROP. IL-PO_2_ contents were 10 and 3 wt % in BMA-1-IL-PO_2_ and BMA-2-IL-PO_2_, respectively. IL-PO_2_ presence during one-pot synthesis also affected the Mg:Al ratio due to pH differences.

ROP of εCL was performed under microwave irradiation. The solvolytically prepared BMA-1 modified with 0.50 wt % catalyst achieved high polymer yields (over 99%) but produced low-molar-weight PCL. The solvothermally prepared nonmodified BMA-2 catalyzed ROP relatively slowly and with a low polymer yield. In contrast, the BMA-2-IL-PO_2_ particles with the immobilized IL exhibited a high catalytic effect for the ROP of εCL, producing PCL with a much higher molecular mass (*M*_w_ up to 74 000 g/mol). Furthermore, the MALDI-TOF MS revealed the formation of three PCL chain distributions differing in end groups, suggesting multiple initiation of ROP by phosphinate anions of IL and by glycerolate and structure-bonded water of BMA. It was finally proved that the solvothermally prepared IL-P-modified BMA acts as both an initiator and a catalyst of the ROP of εCL.

## Supplementary Material



## Notes

Additional SEM images (Figure S1), EDS elemental mapping and analysis (Figure S2, Table S1), original XRPD patterns (Figure S3, Figure S4), TG records (Figure S5), DSC records (Figure S6, Figure S7), and XPS spectra (Figure S8) (PDF)
