# Supplementary material for: Synthesis of Mg–Al Alkoxide/Ionic Liquid Particles as Heterogeneous Catalysts for Ring-Opening Polymerization of ε‑Caprolactone
Source: ACS Omega. 2026 Jul 3;11(28):42352–65. doi: 10.1021/acsomega.6c02540 (PMC13393023; doi:10.1021/acsomega.6c02540)
Supplement: Supplementary file 1 [file ao6c02540_si_001.pdf]

Synthesis of Mg-Al alkoxide / ionic liquid particles as heterogeneous catalysts for ring-opening polymerization of ε-caprolactone

Michal Navrátil[a], Marwa Rebei[b], Darina Smržová[a], Anna Vykydalová[a,c], Petr Bezdička[a], Martin Kormunda[d], Natalija Murafa [a], Zuzana Walterová[b], Lívia Kanizsová[b], Hynek Beneš[b], Petra Ecorchard[a,]*

[a] Institute of Inorganic Chemistry of the Czech Academy of Sciences, Husinec-Řež č. p. 1001, 250 68 Řež, Czech Republic

[b] Institute of Macromolecular Chemistry of the Czech Academy of Sciences, Heyrovského nám. 2, 162 00 Prague 6, Czech Republic

[c] Polymer Institute, Slovak Academy of Sciences, Dúbravská cesta 9, 845 41 Bratislava, Slovakia

[d] Faculty of Science, Jan Evangelista Purkyně University in Ústí nad Labem, Pasteurova 3632/15, 400 96 Ústí nad Labem, Czech Republic

SEM and EDS analysis images

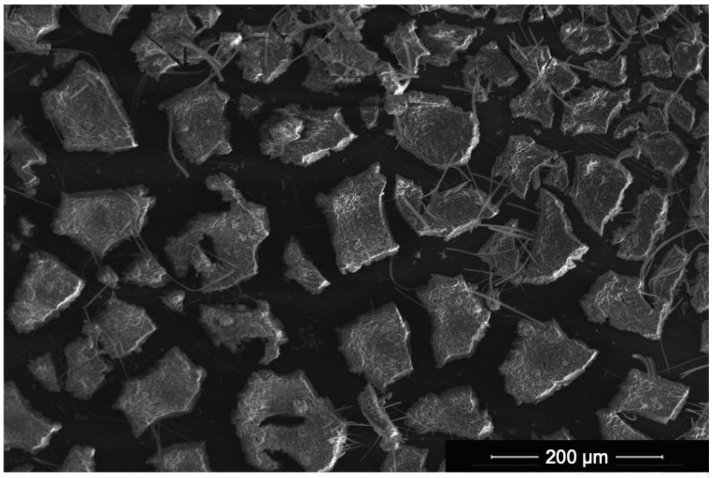

Figure S1: SEM images of BMA-1-IL-PO_2.

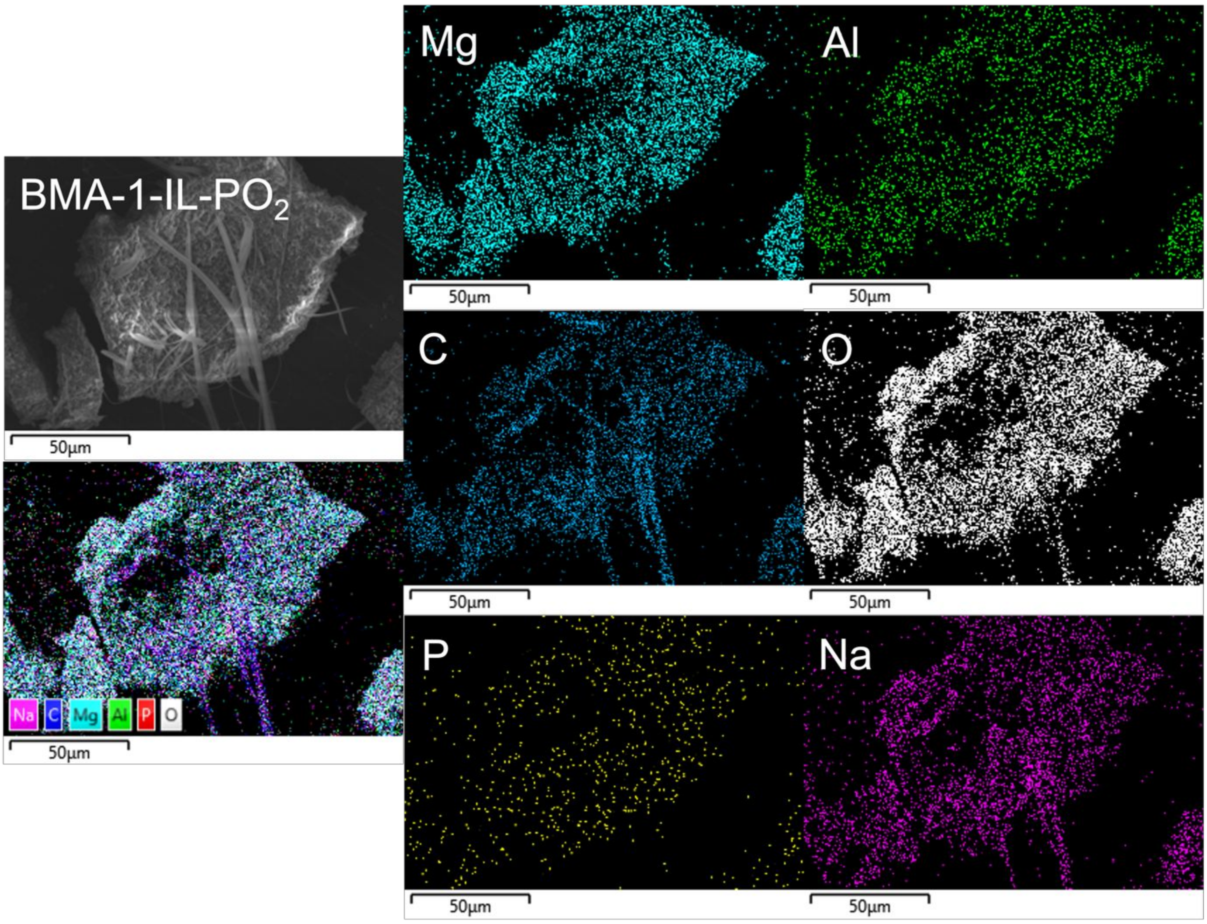

Figure S2: EDS analysis images of BMA-1-IL-PO_2. Mapping and spectra are shown.

Table S1 SEM-EDS analysis of all samples

SEM-EDS	O [at.%]	C [at.%]	Mg [at.%]	Al [at.%]	Na [at.%]	P [at.%]
BMA-1	58.4	24.7	13.8	2.5	0.5	-
BMA-1-IL-PO_2	45.7	37.6	9.1	2.7	4.4	0.6
BMA-2	49.5	36.6	10.1	3.8	-	-
BMA-2-IL-PO_2	49.6	34.1	9.2	6.8	-	0.3

X-ray powder diffraction

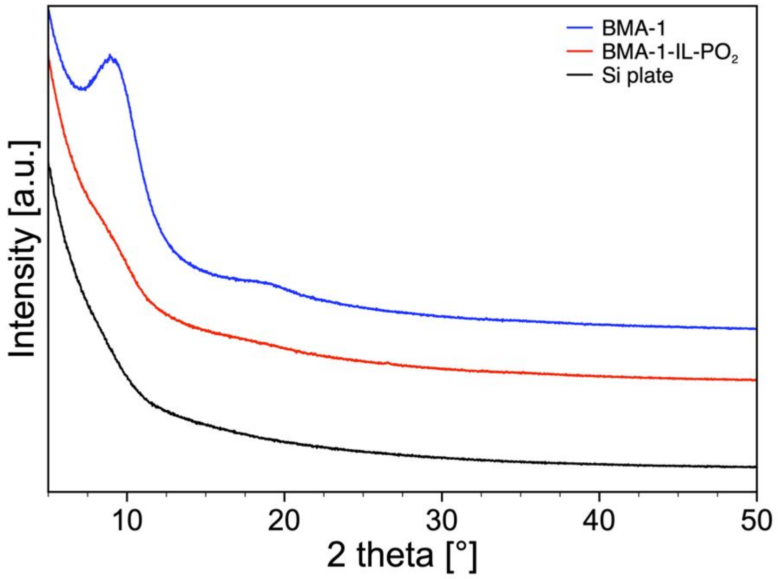

Figure S3: XRPD patterns of solvolytically prepared BMAs measured on silicon plates. The black line represents the background of the silicon zero background holder (after subtraction of background is shown in the manuscript). XRPD pattern after subtraction of background is shown in the manuscript.

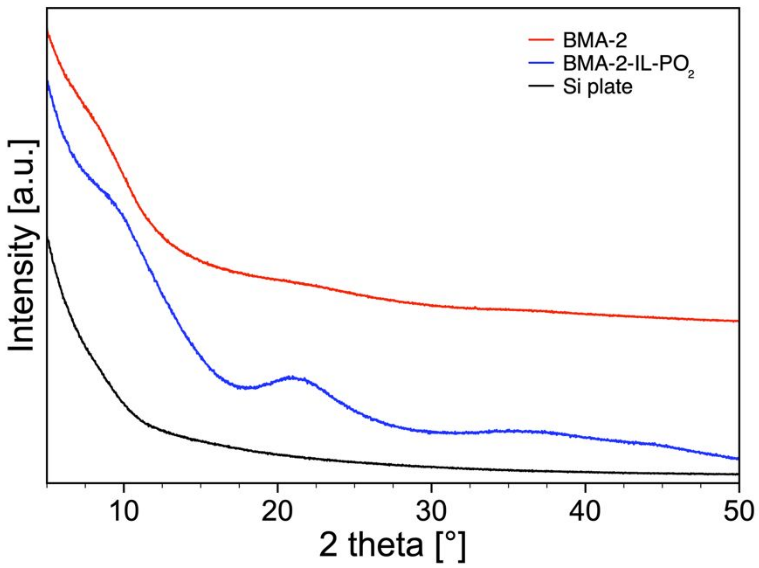

Figure S4: XRPD patterns of solvothermally prepared BMAs. BMA-2 was measured on a Si zero background holder (background represented by the black line), and BMA-2-IL-PO_2 was prepared by front filling. XRPD pattern after subtraction of background is shown in the manuscript.

Thermogravimetric analysis coupled with mass spectrometry and DSC analysis

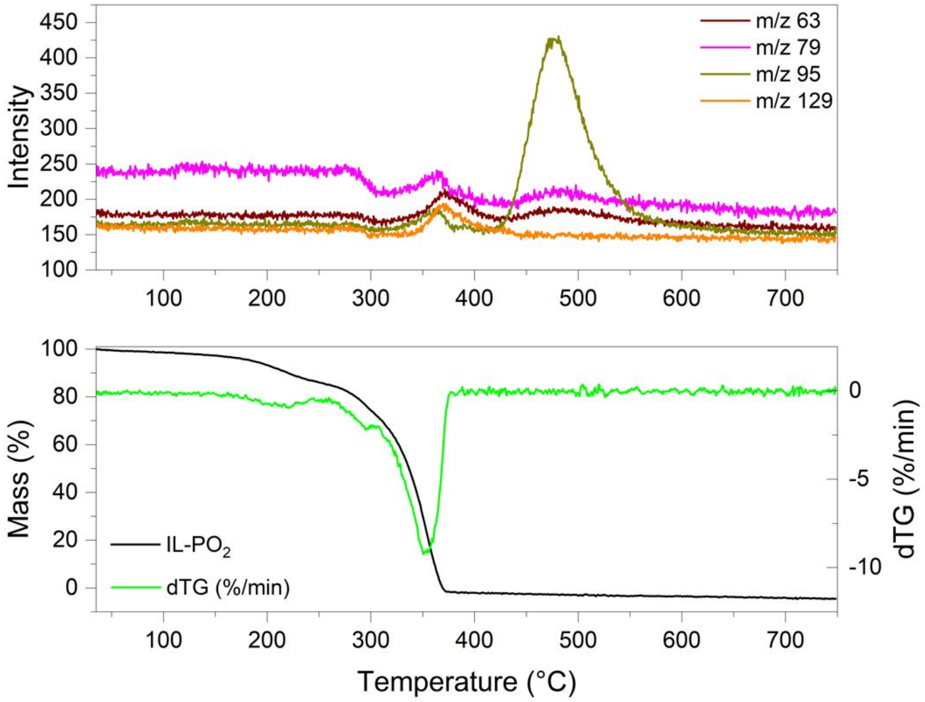

Figure S5: The records of TG, dTG and the chosen values of m/z for IL-PO_2.

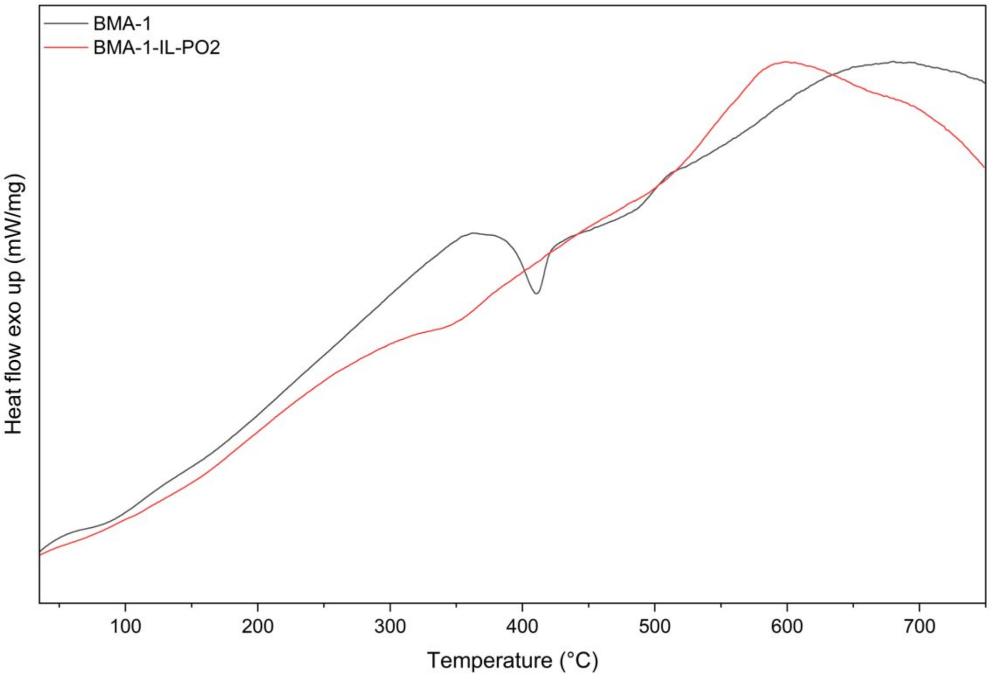

Figure S6 DSC records of BMA-1 and BMA-1-IL-PO_2.

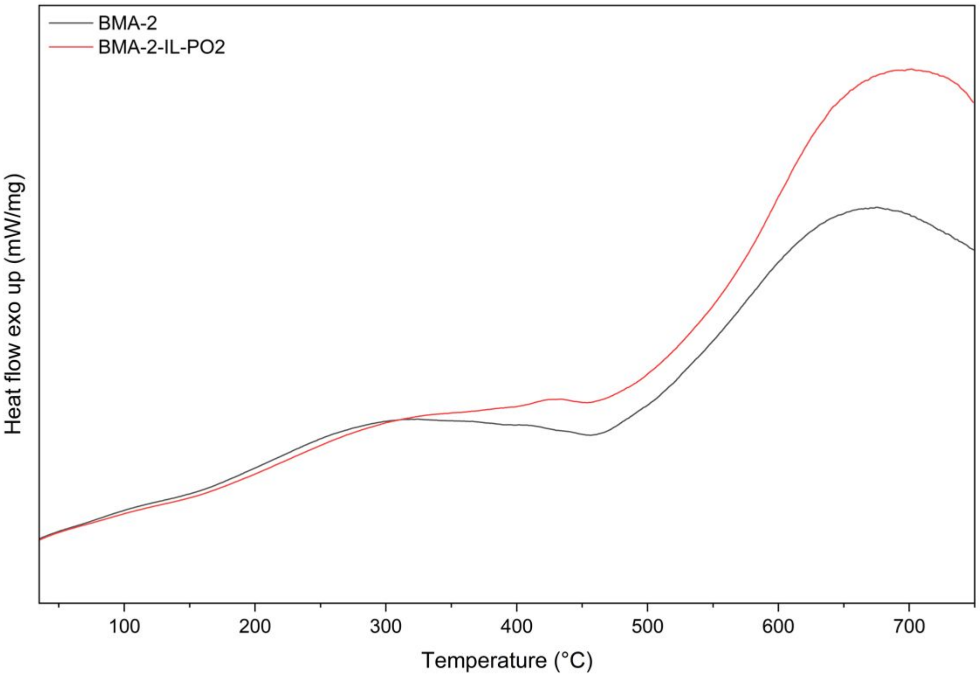

Figure S7 DSC records of BMA-2 and BMA-2-IL-PO_2.

X-ray photoelectron spectroscopy

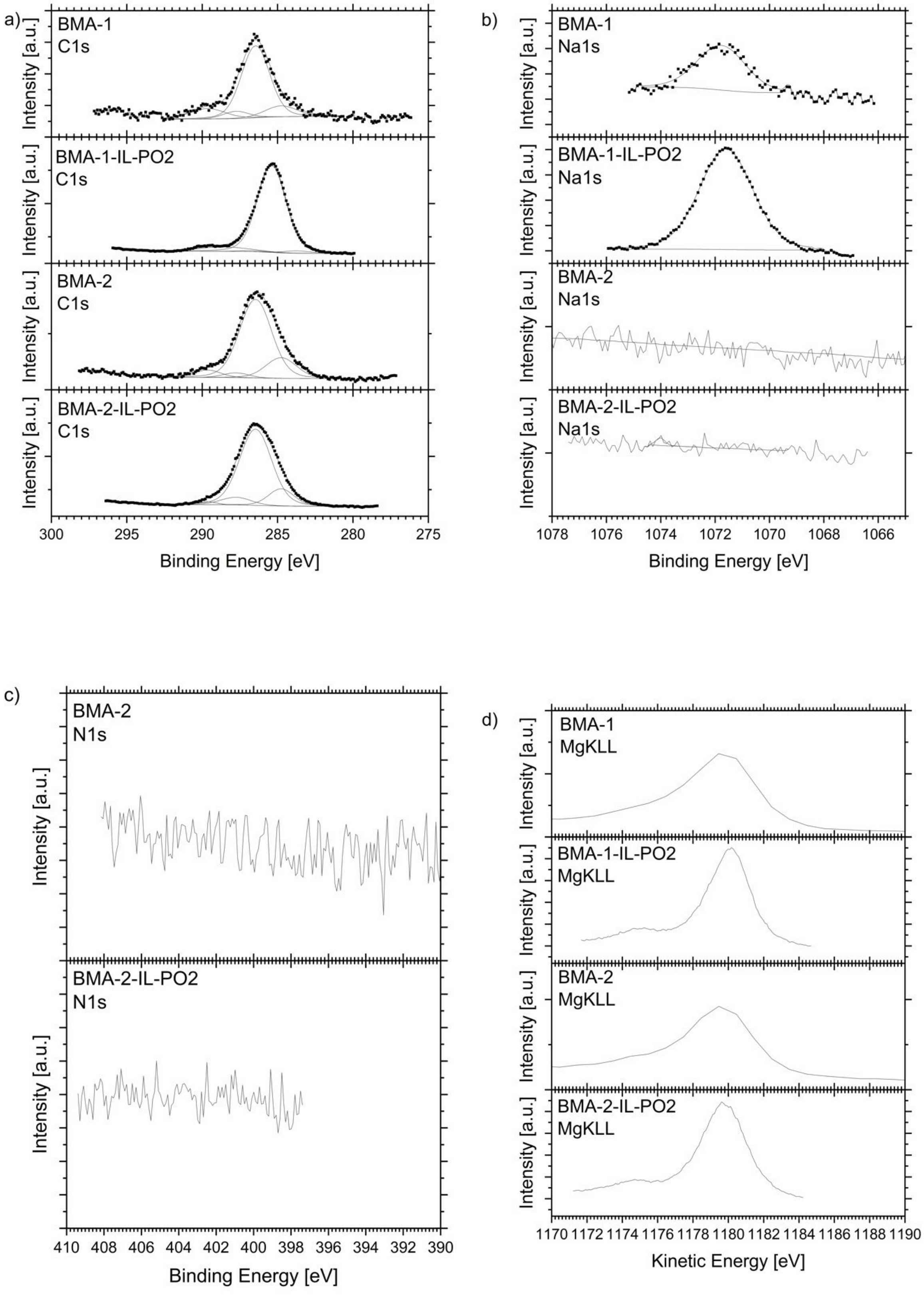

e)

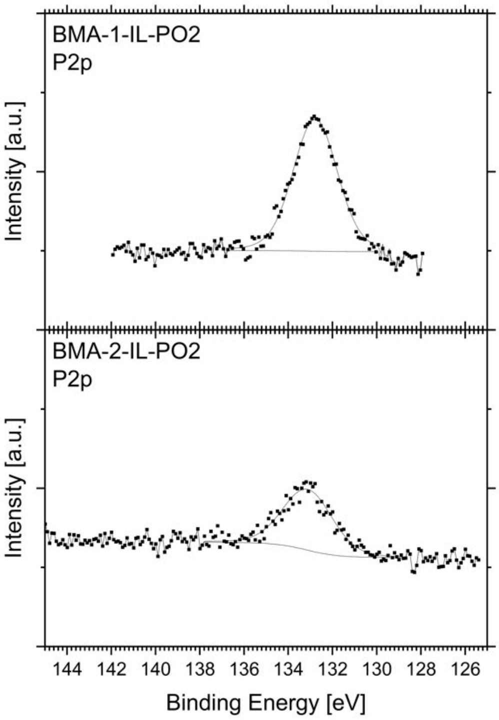

Figure S8 High resolution XPS spectra of a) C1s, b) Na1s, c) N1s, d) Mg KLL, and e) P2p
